# Dairy farm worker milking equipment training with an E-learning system

**DOI:** 10.3168/jdsc.2022-0217

**Published:** 2022-07-14

**Authors:** Valeria M. Alanis, W. Recker, Paula A. Ospina, W. Heuwieser, Paul D. Virkler

**Affiliations:** 1 Departamento de Medicina Preventiva y Salud Pública, Facultad de Medicina Veterinaria y Zootecnia, Universidad Nacional Autónoma de México, Ciudad Universitaria, CDMX 04510, México; 2 Department of Population Medicine and Diagnostic Sciences, College of Veterinary Medicine, Cornell University, Ithaca, NY 14853; 3 Lechear LLC, King Ferry, NY 13081; 4 Clinic for Animal Reproduction, Faculty of Veterinary Medicine, Freie Universität Berlin, 14163 Berlin, Germany

## Abstract

In many farms, the logistics of on-farm training are a limiting factor due to communication challenges in the workplace (i.e., cultural differences, language barriers, impaired listening skills) and limited access to industry professionals. The use of E-learning systems may help to improve communication and can be sensitive to cultural challenges. The objectives of this study were to (1) identify how many of the high-priority problems in the milking parlor relate to milker training in the areas of milking equipment and milking routine, (2) design and test an E-learning training system for dairy farm milkers related to milking equipment, and (3) gain feedback targeted to improve subsequent E-learning training modules. An interactive online training course on basic checks of the milking equipment was developed with a cloud-based authoring software. A total of 95 dairy farm workers on 15 commercial dairy farms in northern New York State (USA) were trained and participated in the study. Milk quality professionals performed an initial evaluation of 3 main areas: equipment analysis, milker assessment, and cow assessment. The 3 most important risk factors for mastitis were summarized for each farm. A training event was scheduled with milkers guided by a bilingual (English and Spanish) professional in milk quality. Over 50% of the farms (8/15) had one or more of the top 3 priorities involving problems with milking equipment, which milkers could have detected and reported to management. All participants completed the module, 95% stated that they felt capable of checking the equipment before milking, and 86% felt more confident in reporting equipment problems to the manager after having taken the course. There were also differences between managers' and milkers' statements on how and whether milking equipment training was offered or not on the farm. This can be explained due to the lack of or secondary to poor communication between managers and employees on training objectives and goals on the farm. Our results also show that milking equipment issues which milkers could detect and report are common on dairy farms and reinforces the need for additional milker training in this area.

## Highlights

• Milking equipment malfunctions, which milkers could detect, are common on dairy farms.
• Our results reaffirm the lack of communication between managers and employees, which restate the necessity to state objectives and goals on every training.
• Practical logistics of on-farm training are a limiting factor, so the use of online training is a reasonable alternative for dairy farms, increasing employees' confidence by providing more detailed training content.

## Graphical Abstract

**Figure:**
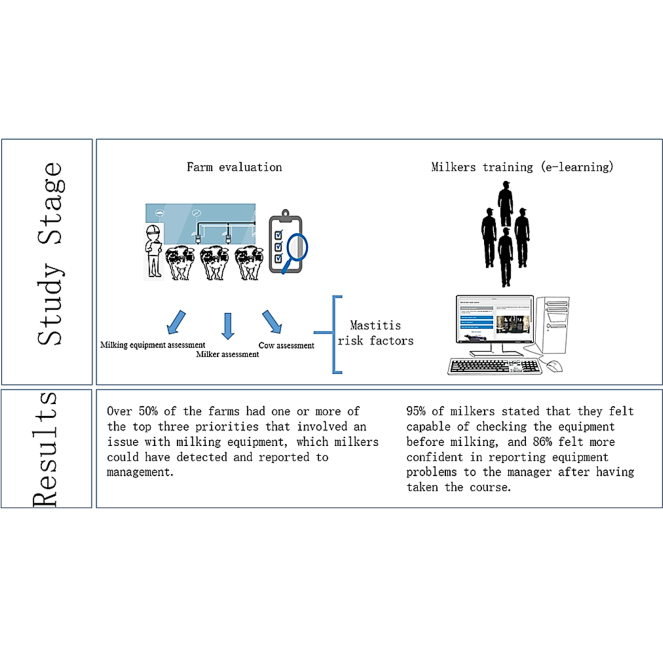
**Summary:** Herd managers and owners are responsible for providing adequate training to their employees to produce high quality milk. Such training should be planned based on farm needs. This study identified how many of the relevant problems, in the milking parlor related to milker training, were present in 3 main areas: equipment analysis, milker assessment, and cow assessment. The study was conducted on 15 commercial dairy farms in northern New York State (USA). Over 50% of the farms had one or more of the 3 most important problems with milking equipment, which milkers could have detected and reported to management. A total of 95 dairy farm workers were trained using E-learning modules related to milking equipment. The use of this type of training isa reasonable alternative for dairy farms, increasing employees' confidence by providing more detailed training content.

Learning is defined as the absorption of information aimed at increasing knowledge, skills, and behaviors by employees to apply it in real-life situations, while training is aimed at facilitating this process and depends on the quality of the transfer of the desired information (Noe, 2017). As in any competitive business, a dairy farm should have both learning and training as key components in its human resource development plan. Hands-on training, demonstrations, discussion, and one-on-one training, with some mixed opinions for online, books, or manuals, are the preferred delivery methods. The next-generation farmers, however, want an increase in online learning resources for meaningful educational experiences and opportunities (Franz et al., 2010; Baugher et al., 2017). There are generally 3 options for training: in-person training by on-farm personnel or industry professionals, access to E-learning systems, which is an educational and training platform that takes place over the internet; and finally a combination of both. Since the mid-1990s, US dairy farms have seen an increase in the number of Latino employees (Jenkins et al., 2009; Menger et al., 2016), which can affect the business through communication and cultural challenges. The latter can increase the risk of work-related injury and illness due to inexperience, limited education, and English/Spanish division between workers and managers (Arcury et al., 2010; Hurley and Lebbon, 2012; Menger et al., 2016). This complicates management-employee relationships and results in more rapid employee turnover (Barkema et al., 2015; Erskine et al., 2015). Twenty-five percent of these Latino employees speak an indigenous language or regional dialect as their native language (Arcury et al., 2010); and Spanish as a second language. Among those who can speak some Spanish, many do not read or write it. The English proficiency among these groups is low. In addition, the education distribution in this group is not homogeneous, ranging from zero years of education to individuals with professional degrees (Maloney et al., 2016; Rodriguez et al., 2018; Sischo et al., 2019). This deficient formal education and the challenge to effectively communicate creates additional barriers such as lack of confidence in their ability to learn, limited access to training tools, and a reduced likelihood to perform managerial functions compared with their English-speaking counterparts (Stack et al., 2006). Recently published studies, however, showed that employers oftentimes underestimate the employee's interest in learning and commitment to the success of the farm (Durst et al., 2018) and that the absence of training or training materials negatively affects employee recruitment and retention (Moore et al., 2020). For that reason, training should include bilingual content, as it has been demonstrated that this benefits the employees (Chase et al., 2006; Raymond et al., 2006; Rovai et al., 2016).

Achieving or maintaining high standards of milk quality relies heavily on dairy employees. Therefore, milkers should be well trained and conscientious about milking routines and milking equipment. Few studies have focused on how milking training is provided in the United States, where instructor-led training is most common. The majority of employees, however, stated that training of milking routines is provided by fellow employees or not at all (Erskine et al., 2015; Rovai et al., 2016). Lack of training has been claimed to be one of the main reasons for lower detection of animal health problems, poor animal handling and management of calving events, and poor milking routines (Gutierrez-Solano et al., 2011; Schuenemann et al., 2013). Farms with frequent training of milking personnel achieve faster milking speeds and lower rates of clinical mastitis (Rodrigues et al., 2005).

Although most dairy producers and industry professionals would agree that both initial hire and ongoing employee training are essential to assuring proper adherence to protocols (Jansen et al., 2010; Erskine et al., 2015; Belage et al., 2019), the practical logistics of on-farm training are a limiting factor. This has been especially true during the COVID-19 pandemic, which has severely limited in-person training and revealed opportunities for new ways to deliver training. The use of online information has become a part of normal life worldwide and it seems reasonable to incorporate it as a training tool in dairy farms. In addition, online training has been shown to be effective at creating a feeling of confidence and accuracy in work performance (Hesse et al., 2019) and is an effective way to deliver safety awareness training to dairy employees (Rodriguez et al., 2018). The objectives of this study were to (1) identify how many of the high-priority problems in the milking parlor relate to milker training in the areas of milking equipment and milking routine, (2) design and test an E-learning training system for dairy farm milkers related to milking equipment, and (3) gain feedback targeted to improve subsequent E-learning training modules.

An interactive online training course on basic checks of the milking equipment was developed by the authors of this study with a cloud-based authoring software (GomoLearning). The course consisted of 5 modules (i.e., liner alignment, checking vents, checking pulsators, assessing vacuum levels, and preparing the milk house) and was available both in English and Spanish. Each module took the user 6 to 8 min to complete with an overall contact time for all 5 modules of approximately 30 to 40 min. The modules were designed to be user-friendly and straightforward to ensure an engaging and nonintimidating learning experience. The content was displayed in a step-by-step fashion based on images and videos. Textual information was reduced to a minimum and aimed at how to perform each step of the equipment check, why each check is important to udder health, and the quality of the milk produced. In 2 of the modules, there was an option for the participant to choose between reading the texts and listening to audio. Four sets of 3 questions each were embedded into the modules to collect data on the background information of the participants, the farm, and perceptions of the modules. At the end of each module, there were quiz questions to gauge how well the participant understood the main concept. An introductory module was provided to familiarize the participant with the major components of the milking system and explain the function of each component. This section was designed for employees with limited milking experience. Participants could choose to explore each component on their own or have it taught to them through a narrated video. A glossary of relevant key terms in the modules was available throughout including images and brief definitions.

After completion, a β-testing was conducted by 2 of the authors (VA, PV) on 1 dairy farm with Spanish and English employees with the goal to identify and correct errors and malfunctioning before the field study. Next, the modules were used in a field study conducted on 15 commercial dairy farms across 4 counties in northern New York state between September 2020 and January 2021. These farms were a convenience sample based on long-term working relationships with the Quality Milk Production Services (**QMPS**) from the Animal Health Diagnostic Center at Cornell University and the willingness of the farm to participate in this research. The main breed of cattle on these farms was Holstein-Friesian, with an average farm size of 800 cows (ranging from 250 to 2,400) and an average milk production of 38.6 kg/cow per d (ranging from 32.7 to 49.1). Monthly mean SCC was 178,000 cell/mL (ranging from 100,000 to 310,000 cells/mL). The average number of milkers per farm was 6 (ranging from 3 to 14).

For each of the farms, an initial extension survey was performed to assess the risk factors for mastitis. An extension survey is a service provided by QMPS that is often used by dairy producers who have large herds, whereby milking procedures, management, housing, equipment, and mastitis control are evaluated. This service is performed by QMPS professionals, including veterinarians and skilled technicians that have wide experience in developing specific programs aimed at mastitis prevention, parlor and milking equipment operation, and quality milk production. The information is then used to make recommendations for improved management and mastitis control. Each extension survey consisted of an assessment of 3 main areas: (1) equipment analysis involving average claw vacuum, milk line vacuum during milking, and graphing all pulsators, (2) milker assessment involving milking routine, milk flow rate analysis, unit alignment scoring, teat end cleanliness scoring, and dip coverage, and (3) cow assessment involving teat scoring, strip yields, udder hygiene scoring, and an assessment of the environment.

During equipment analysis, the average claw vacuum at peak flow and the pulsation parameters were measured with the unit on the cow and milk flowing through the claw per the National Mastitis Council (**NMC**) guidelines for dynamic testing (NMC Procedures, 2012). Milkline vacuum stability was assessed per NMC guidelines over an approximately 30-min period at the milk inlet closest to the receiver jar during normal milking operations. All pulsators were also tested statically per NMC guidelines.

For milker assessment, milking routine timing focused on initial stimulation time, pre-dip contact time, and the lag time from stimulation to unit attachment. The milk flow rates of individual cows were measured using an electronic milk flow meter (Lactocorder, WMB). Unit alignment was measured using a 2-category scoring system (proper or improper unit alignment) and was assessed within the first 2 min after unit attachment, with any 3-quarter cow not scored. Teat end cleanliness assessment was performed after teat preparation but before unit attachment. A 10 × 10 cm gauze soaked in alcohol was used to swab the teat end. The scores were recorded using a 1- to a 4-category system (Cook and Reinemann, 2007). Dip coverage was assessed by visually observing all surfaces of the teat including the teat end and all sides of the teat barrel and evaluating whether or not dip was present.

For cow assessment, teat scoring was performed within 1 min of unit detachment using the Teat Club International scoring system (Mein et al., 2001). At least 20% of the lactating cows were scored in the categories measuring the short- and long-term effects on the teats. Strip yields were performed immediately after unit detachment. Each teat was stripped for a maximum of 15 s and the total volume of milk from all 4 teats recorded. Udder hygiene was scored using a 1- to 4-category system (Schreiner and Ruegg, 2002). The environment was assessed by walking the lactating cow stalls and scoring in the categories of cleanliness, bedding levels, and cow positioning.

Based on the data from the extension survey, the risk factors for mastitis were summarized and ranked by importance for each farm. The 3 most important risk factors for each farm were then placed into the following categories: milking equipment malfunction, equipment malfunctions that could be detected by milkers, inadequate milking routine, and other. After the extension survey, a 1-h training event was scheduled with milkers in each farm to apply and test the online course in a real-life situation. Milkers, and farm managers or owners, completed an initial written questionnaire at the beginning of each session regarding background and training on farm.

As the online course can be run on any web-enabled device, milkers were asked either to use their cellphones or a tablet that we provided. Each participant received a unique login ID based on a random number and linked to the farm to ensure anonymity and create a safe working environment for the milkers. A description of the terms and conditions of use and the privacy and data protection policies was provided on the first page of the course. This training project was performed following the oral consent guidance from the Cornell University Institutional Review Board for Human Participants.

Milkers were asked to complete the 5 modules during the training event, which provided a 10-min introduction to the course. Any person with reading disabilities was guided by a bilingual project collaborator and any question regarding the content was clarified when necessary. All milkers that completed the modules received a printed certificate with their name on it. Oral feedback was collected about how they felt about the module, and if they had suggestions for improvements.

Based on the extension survey results, 14 of 15 farms had a milking equipment problem as at least one of the top 3 risk factors for mastitis. Eight farms had one or more of the top 3 risk factors that involved milking equipment malfunction, which milkers could have detected and reported to management. Inadequate milking routines also accounted for a large portion of the risk factors with 13 of the farms. For the category of equipment malfunctions that milkers could have detected or corrected, pulsators and the use of the manual button (canceling the automatic cluster remover) accounted for 36% (4 out of 11) and 27% (3 out of 11), respectively. A short lag time from stimulation to attachment (47%, 7 out of 15) and teat end cleanliness (40%, 6 out of 15) were the most common problems found in the inadequate milking routine category (Table 1). Within the category of milking equipment malfunction the higher proportion was related to automatic take-off (**ATO**) adjustments (40%, 6 out of 15), and pulsator function or adjustments (33%, 5 out of 15).

**Table 1:** Priorities based on extension surveys^1^ used to identify mastitis risk factors in 4 main areas: milking equipment malfunction, equipment malfunctions that could be detected by milker, milking routine errors, and other issues^2^ in 15 commercial dairy farms in northern New York state

Farm	Number of milkers	Milking equipment malfunction	Equipment malfunctions that could be detected or corrected by milker	Inadequate milking routine	Other issues
A	6	Pulsator adjustment ATO^3^ adjustment	Not detected^4^	Lag time too short	Not detected
B	5	Pulsator adjustment	ATO not functioning	Lag time too short	Not detected
C	6	Pulsator function	Units on manual	Poor use of unit alignment device	Not detected
D	5	Units on manual	Units on manual	Teat end cleanliness	Not detected
E	5	Not detected	Shut-offs not working Plugged vents	Lag time too short	Not detected
F	14	ATO adjustment	Pulsators not working	Lag time too long	Not detected
G	7	ATO adjustment	Pulsators not working	Lag time too short	Not detected
H	3	Pulsator adjustment	Not detected	Teat end cleanliness	Bedding levels
I	7	System vacuum incorrect	Not detected	Lag time too short Teat end cleanliness	Not detected
J	6	Not detected	Units on manual Pulsators not working	Lag time too short	Not detected
K	8	Not detected	Not detected	Teat end cleanliness Pre-dip coverage	Bedding levels
L	10	System vacuum incorrect ATO adjustment	Not detected	Not detected	Bedding levels
M	7	Not detected	Units not retracting correctly	Teat end cleanliness	Teat skin condition and postdip
N	3	ATO adjustment Unit alignment devices	Pulsators not working	Not detected	Not detected
O	3	System vacuum incorrect ATO adjustment	Not detected	Teat end cleanliness	Not detected

1 Service provided by Quality Milk Production Services (QMPS) from the Animal Health Diagnostic Center at Cornell University. The QMPS professionals, including veterinarians with parlor experience and skilled technicians, evaluate management, milking routine, housing, equipment, and mastitis control.

2 Issues not related to milking equipment or milkers' performance that could have an effect on mastitis risk.

3 ATO = automatic take-off.

4 Not detected = no related deviations or malfunctions detected during extension survey.

A total of 95 milkers participated in this study, with 90 and 5 of these milkers having Spanish and English as their native language, respectively. From the initial questions, almost half (46%) of the milkers had not milked cows before starting to work at this farm, and 40% had worked less than 6 mo on the current farm. Milkers who had been at the farm more than 6 mo stated that the last time they had received training on the farm was more than 6 mo ago, and 83% had received some type of training when they started the position. According to the written questions, 67% of the milkers stated that they had received milking equipment training when they had started the position. In 59% of the cases, this training was conducted by another milker. This could be counterproductive, due to distortion of information relevant for a given task leading to protocol drift. Seven farms indicated that they provided milking equipment training to their milkers. However, there were some discrepancies on 4 of those farms, as at least one of the milkers contradicted this statement. On the other hand, on 3 out of the 8 farms that indicated they did not provide such training, at least 80% of the milkers in each farm contradicted this. This can be explained as a lack of agreement between managers and employees on what training is with respect to milking equipment. About 69% of milkers reported that they were expected to fix milking equipment problems on their farms, but 45% were not trained at all or were not satisfied with their training in milking equipment (Table 2). As for hours per pay period, 77 milkers reported less than 80 h, 7 milkers 80 to 130 h, and 10 more than 130 h. One milker did not mark any option.

**Table 2:** Anonymous responses (no.; % in parentheses^1^) from 95 milkers in 15 commercial dairy farms in northern New York state concerning training and milking equipment

Question	Milkers
Have you ever had training on the milking equipment? (How it works? What to do if it breaks?)	
Yes	64 (67.4)
No	28 (29.5)
Not sure	3 (3.1)
Training provided in milking equipment	
Employee and manager responses coincided	64 (67.4)
Employee and manager responses differed	31 (32.6)
Are you satisfied with that training?	
Yes	68 (71)
No	18 (19)
Not sure	9 (10)
Who trained you on the equipment on this farm?	
Another milker	56 (59)
Manager	12 (13)
External professional	12 (13)
Nobody	10 (11)
Other	5 (5)
How many hours do you work per pay period?	
Less than 80 h	77
More than 80 but less than 130 h	7
More than 130 h	10
No answer	1

1 Due to rounding, percentages do not always add up to exactly 100%.

All milkers completed the online course and 9 relaunched it after completion. This completion rate was much higher compared with a previous study (only 6%, P. Virkler, unpublished data) in which milkers worked at their own discretion and in their leisure time. This shows mere availability online was not sufficient to complete an entire training tool. Instead, it seems important to provide the employees a dedicated time to complete the training, where the session is conducted at a pre-specified date and time and supported by the farm management. Interestingly, 70% of the milkers stated a preference for the text compared with the audio recordings. However, 73% of the milkers reported that they wanted audio recordings in future training. One explanation for this discrepancy is that the participants were reluctant to admit a limited reading proficiency for the online course and therefore opted for audio recordings in future materials.

We did not conduct a before-and-after training comparison, but the questions after each module were answered correctly in more than 80% of the cases in 3 of the 5 modules, and 75% in the milk house module. Unfortunately, due to a data recording issue, the percentage that answered correctly was not available for the vents module. At the end of the training, 95% and 87% of the milkers stated that they were able to test the equipment before milking, and they were confident to communicate milking equipment-related issues to the manager, respectively. We hypothesize that the basic concepts learned will motivate the milkers to perform the skills covered and improve the reporting of milking equipment problems (Table 3).

**Table 3:** Responses (no.; % in parentheses^1^) from 57 participants who completed a survey embedded in an E-learning module on milking equipment in 15 commercial dairy farms in northern New York state

Question	Milkers
Do you feel confident now to tell the management that there is an equipment problem?	
Yes	49 (86)
No	3 (5.3)
Not sure	5 (8.8)
After this training, are you able to check the equipment before milking?	
Yes	55 (95.6)
No	0 (0)
Not sure	2 (4.4)
Would you like to have audio recording in future training modules?	
Yes	41 (70.7)
No	5 (8.6)
Not sure	11 (19.0)

1 Due to rounding, percentages do not always add up to exactly 100%.

Our results show that milking equipment issues were common on these 15 dairy farms and reinforce the need for additional milker training in this area. Even though a certain percentage of milkers reported some previous training, the percentage of milkers who were not trained at all, were not satisfied with the training, or were unsure if they were trained or satisfied was considerable (45%). This finding shows an opportunity for efficient training materials that employers could use for new employees to train them on how to detect milking equipment problems. This could be particularly valuable because many of the incoming employees are lacking at least basic knowledge or skills from growing up on a farm or from previous working experience on a farm (Hagevoort et al., 2013; Erskine et al., 2015).

All herd managers agreed on the need for training tools to better educate their employees on milking equipment. Nonetheless, only one of the herd managers launched the module, but failed to complete it. This is an interesting finding because supervisor support is crucial to training effectiveness and plays an important role in motivation (Chiaburu and Tekleab, 2005), which can increase employee engagement. Farm owners were willing to pay employees to be trained in a dedicated session; however, just giving milkers time to complete training at their own initiative was not successful (P. Virkler, unpublished data). This observation may also demonstrate the importance of providing owners with a systematic approach to training and training materials to establish a training culture with systematic and continuous training for milkers.

This E-learning option may help to simplify training by providing access to more remote areas through online learning approaches. Further research is warranted to demonstrate that online training is effective in improving measurable outcomes related to milking equipment issues. This could be achieved by adding a follow-up assessment to test certain manual skills or routines. This would allow a herd manager to determine whether a milker has retained the knowledge and adequately learned the new skills, as well to implement it correctly.

Even though this is a small study, farms were a convenience sample, and the majority of employees were non-English speakers, it provides insightful information in an under-researched area. It also substantiates the importance of efficient employee-manager communication. A limitation of our study is the missing comparison of a pre- and postassessment. Further research is warranted to measure whether participants truly applied what they learned through an online training. This study provides more evidence that there is a lack of a learning culture on some farms and a lack of a structured training program. More work is needed to help farms realize the importance of developing a learning culture where training and feedback are provided to milkers regularly to promote continuous improvement and job satisfaction.
